# Identifying the Effect of Cognitive Motivation with the Method Based on Temporal Association Rule Mining Concept

**DOI:** 10.3390/s24092857

**Published:** 2024-04-30

**Authors:** Tustanah Phukhachee, Suthathip Maneewongvatana, Chayapol Chaiyanan, Keiji Iramina, Boonserm Kaewkamnerdpong

**Affiliations:** 1 Computer Engineering Department, Faculty of Engineering, King Mongkut’s University of Technology Thonburi, Bangkok 10140, Thailand; tustanah.p@mail.kmutt.ac.th (T.P.); suthathip.man@kmutt.ac.th (S.M.); chayapol.chekl82@mail.kmutt.ac.th (C.C.); 2 Graduate School of Systems Life Sciences, Kyushu University, Fukuoka 819-0395, Japan; iramina@inf.kyushu-u.ac.jp; 3 Biological Engineering Program, Faculty of Engineering, King Mongkut’s University of Technology Thonburi, Bangkok 10140, Thailand

**Keywords:** cognitive motivation task, electroencephalography (EEG), motivation, temporal association rule mining (TARM)

## Abstract

Being motivated has positive influences on task performance. However, motivation could result from various motives that affect different parts of the brain. Analyzing the motivation effect from all affected areas requires a high number of EEG electrodes, resulting in high cost, inflexibility, and burden to users. In various real-world applications, only the motivation effect is required for performance evaluation regardless of the motive. Analyzing the relationships between the motivation-affected brain areas associated with the task’s performance could limit the required electrodes. This study introduced a method to identify the cognitive motivation effect with a reduced number of EEG electrodes. The temporal association rule mining (TARM) concept was used to analyze the relationships between attention and memorization brain areas under the effect of motivation from the cognitive motivation task. For accuracy improvement, the artificial bee colony (ABC) algorithm was applied with the central limit theorem (CLT) concept to optimize the TARM parameters. From the results, our method can identify the motivation effect with only FCz and P3 electrodes, with 74.5% classification accuracy on average with individual tests.

## 1. Introduction

Motivation is an essential state of mind that can enhance the attention and performance of learners during their learning process. With the advantages of being motivated to perform the task, many researchers have taken an interest in studying motivation. In educational psychology, motivated individuals were found to have preferable traits that enhance their learning performance [1,2,3,4,5]. Renninger and Wozniak [1] studied the motivation of children to the item of interest. They found that the high level of motivation the children felt for the item contributed to increased attention, recognition, and recall performance. Various studies [2,3,4] found that the motivation of the participants to demonstrate their competence positively correlates with the actual achievement of the participants. This type of motivation often occurs when they compare their performance to that of others. In 2022, Mussel [5] researched the curiosity of students, which is a factor leading to motivation. Their study’s results suggested that curiosity is significantly related to the student’s academic performance.

In studies [1,2,3,4,5], motivation could occur from various motives, such as interest [1], reward (grade, emotional self-rewarding) [2,3,4], or even curiosity [5]. Each of these motives is a factor that can motivate us to perform the task. These motives affect different areas within our brain related to their activities. Various fMRI studies [6,7,8] have reported the relationship of the brain areas and their activities related to these motives. Lee et al. [6] studied the effects of intrinsic and extrinsic motivation. They found that reward (extrinsic motivation) affects the right posterior cingulate cortex, which was hypothesized to be a reward-based area. On the other hand, self-satisfying feelings (intrinsic motivation) were found to affect the right insular cortex, which was hypothesized to be an emotion-based area. Ulrich et al. [7] studied the motivation resulting from the challenge of the task. Their research suggested various areas related to the process of motivation, including the putamen, related to the coding of increased outcome probability (probability to choose higher reward outcome); the inferior frontal gyrus (IFG), related to a deeper sense of cognitive control; the medial prefrontal cortex (MPFC), related to the decreased self-referential processing; and the amygdala (AMY), related to the process of decrease in negative arousal. The study of curiosity and interest by Lee and Reeve [8] found that the anterior insular cortex (AIC) and striatum work together in an intrinsic motivation system. While AIC is related to the subjective feelings resulting from the body, the striatum plays a crucial role in reward processing. The striatum is also a central part of extrinsically generated motivation. They also found a relationship between intrinsic motivation and the frontal areas of the brain (e.g., dorsolateral prefrontal cortex, medial frontal gyrus), which is believed to be related to higher-order cognitive processes.

The fMRI studies [6,7,8] help us understand various brain areas and their activities related to different motives. However, for use in real-world situations, fMRI is inflexible as the measurement due to the limitations of the equipment. EEG, which has advantages in the combination of low cost and flexibility for use in the real-world environment, has become one of the conventional equipments in motivation studies. These EEG studies [9,10,11,12] also found similar results to fMRI studies that motivation could affect various areas depending on the motives. The study of van der Ven et al. [9] investigated the N400 ERP component of EEG on a reading task with the reward depending on the result. They found that C3, Cz, C4, CP1, CP2, P3, Pz, and P4 from the central and parietal areas are electrodes influenced by motivation. The motivation resulting from the challenging task was studied with the mean amplitude of stimulus-preceding negativity (SPN, an ERP component) by Ma et al. [10] in 2017. They found that the challenging level of the task influenced F4, F6, F8, FC4, FC6, and FT8 of the frontal area electrodes. Jin et al. [11] researched interesting/boring tasks with P300 and feedback-related negativity (FRN) ERP components. They found a difference between the interesting and boring cases in F1, Fz, F2, FC1, FCz, FC2, C1, Cz, and C2 electrodes with the FRN and C1, Cz, C2, CP1, CPz, CP2, P1, Pz, and P2 electrodes with the P300 component. In 2018, Brydevall et al. [12] studied an information-seeking task, a curiosity-based task, with a feedback-related negativity (FRN) ERP component. In their study, Fpz, AFz, Fz, FCz, and Cz electrodes were found to be influenced by motivation.

Although EEG can also be used to detect various motivations relating to the learning process [9,10,11,12], different motives leading to motivation affect electrodes in various brain areas. In a real-world situation, such as in the classroom, we could not know the type of motive that each learner has at a different part of the lesson. If we measure the effect of motivation from all known motives, a high number of electrodes would be required. However, for educational purposes, the type of motives may not be significant enough to know; only the motivation effect on brain activities that lead to learning performance is required. Since motivation could affect task performance, we have an idea to investigate the motivation effect through the relationship among the brain areas associated with the task performance affected by motivation (e.g., attention and recognition in the cognitive motivation task). Even though the motive cannot be identified by this approach, the number of electrodes required for measuring the effect of motivation could be reduced.

The cognitive motivation task was used as an example and was focused on in this study. Therefore, the effect of motivation on the relationship between attention and memorization brain areas was analyzed. Attention and memory tasks related to motivation were studied by Robinson et al. [13]. In their study, attention was measured by response time with the Attentional Network Test (ANT), while the Newcastle Spatial Memory Test (NSMT) was used to measure memory. The extrinsic motivation was known when the reward was given, while the Intrinsic Motivation Inventory (IMI) questionnaire was used to measure intrinsic motivation. With these measurements, they found that extrinsic and intrinsic motivation improves the participant’s memory and attentional performance. Their results confirmed that motivation is related to both the attention and memory performance of participants. Additionally, the relationship between attention and memory was found in a top-down process in the case of the successful formation of episodic memories [14,15]. Episodic memory is related to the process of remembering the spatial and contextual features of the visual scene stimulus. In this top-down process, the activation of the area within the prefrontal cortex (attention-related area) leads to the activation of the area within the parietal cortex (memory-related area).

The results of our previous study [16] on the cognitive motivation task with EEG also confirmed the relationship of motivation with attention and memory in the study of Robinson et al. [13]. In our previous study, the participants could freely decide whether they wanted to remember the presented scenic stimulus. The participants were considered as being motivated when they selected that they wanted to remember the stimulus and not being motivated if otherwise. The recognition test was conducted afterward to confirm the results of their motivation for each stimulus. It was found that when the participants were motivated to remember the stimuli, there was a significant difference in attention and memorization-related areas between the cases where they could and could not remember the stimulus afterward. We found a longer continuous alpha desynchronization pattern in the “*being motivated and remembered*” case than in the “*being motivated but forgot*” case. The areas of interest are mainly around the frontal (attention-related area [17]) and left parietal (memory encoding-related area [18]) part of the head, which will be represented by FCz and P3 electrodes in this study. No difference was found among “*not being motivated*” cases where the participants were not motivated to remember the stimulus. The results suggest that motivation can affect both attention and memorization [13,16] and that the occurrence of attention brain activity leads to memorization brain activity [14].

In this study, we further our investigation. We hypothesized that the temporal relationship of brain activities between attention and memorization-related areas could identify the effect of motivation on remembering the stimulus. With this hypothesis, the number of electrodes is reduced to two, which includes the FCz and P3 electrodes. To find the temporal relationship between two brain areas, the concept of Temporal Association Rule Mining (TARM) [19], which is the idea of finding association rules or patterns between two sequences while considering time constraints, could be useful. The TARM concept has been successfully applied to various similar applications [20,21]. For example, in 2009, Hojung et al. [20] tested a TARM-based method with the Saccharomyces cerevisiae cell cycle time-series microarray gene expression dataset and found effective rules for the KEGG cell-cycle pathway. In the field of intelligent transportation systems, Feng et al. [21] proposed a hybrid temporal association rule mining method to predict traffic congestion in a road network. Their experimental results showed high accuracy in the prediction of traffic congestion levels.

We proposed a method based on the TARM concept to identify the motivation effect from the temporal relationship of brain activities between attention and memorization areas while the participants are being motivated. To determine the complex brain activity relationship, the metaheuristic algorithms could be used to optimize the parameters of the method. In this study, we employed an Artificial Bee Colony (ABC) [22] algorithm as an example of the metaheuristic algorithm. The ABC algorithm is known for its performance and simplicity; ABC requires relatively fewer algorithm parameters to adjust than other metaheuristic algorithms while performing well on a variety of benchmark functions. The concept of the Central Limit Theorem (CLT) [23] was applied to identify a suitable representative of the method parameter set. The knowledge contributed by our work not only validates the previous knowledge [13,14,15,17,18] but also provides a method to measure the effect of motivation with a reduced number of EEG electrodes. This contribution could improve future related studies and applications to be more suitable learning services on the individual level (personalized learning services). The knowledge from this study can be applied to help reduce the cost of equipment and improve the flexibility of measurement methods in the real-world environment while also lessening the burden on the users during their learning process in future related studies as well as educational applications.

This article comprises four sections. Section 2 describes the raw data, experiment setting, and cognitive motivation identification method based on the TARM concept. Section 3 provides the results, including the suggested optimized parameter set, accuracy of the constructed model, feature analysis, and model validation. Finally, Section 4 discusses the study’s findings, limitations, and conclusion.

## 2. Materials and Methods

### 2.1. Raw Data

To identify the effect of motivation that could lead to remembering the stimulus, the EEG data from the motivation task are required for the analysis. The data used in this study are identical to those used in our previous cognitive-motivation study [16]. More information on our publicly available dataset can be found in the “Data Availability Statement” section. The participants comprised fourteen male and two female Asian volunteers between 21 and 37 years of age. None of the participants have prior visual perception or memory disorders. The data in this study were obtained from the cognitive-motivational task that was separated into two parts: the cognitive experiment and the recognition test. During the cognitive experiment, the Nihon Kohden Neurofax EEG-1100 equipment (NIHON KOHDEN CORPORATION, Tokyo, Japan) with 32 electrodes was used to measure EEG data with a sampling frequency of 500 Hz. The experiments were conducted in a room with no distractions. All experimental procedures and purposes were disclosed to the participants before the experiments.

In the cognitive experiment, participants were presented with 250 random, unique visual scenic stimuli, one by one. Each stimulus was presented for 3 s. Then, the participant had to decide whether they wanted to remember the stimulus within 9 later seconds. With this setup, the cognitive experiment was completed in around 50 min for each participant. This estimation excludes the setup time and the short break requested by participants. The participants could freely make their decision on whether they wanted to remember the scene. The decision was used as an indication of their motivation; the trial is considered a “*being motivated*” case when the participant chooses to remember the stimulus and a “*not being motivated*” case if otherwise. Because motivation is the topic of the study, it depends on the participants’ motivation towards the stimulus; hence, the number of trials between two cases could be unequal for each participant.

In the recognition test, 500 random scenes, comprising 250 from the cognitive experiment and 250 new scenes, were presented. The participants were asked to answer whether they recognized the presented scene in the cognitive experiment. The answer from this test indicated the motivation effect corresponding to their motivation for the stimulus in their prior cognitive experiment. There are no time constraints in this recognition test. The brain signals were not measured during the recognition test.

By relating the motivation of the participant to the corresponding results from the recognition test, the data were categorized into four groups: “*being motivated and remembered*”, “*being motivated but forgot*”, “*not being motivated but remembered*”, and lastly, “*not being motivated and forgot*”. Since the purpose of this study is to analyze the effect of motivation, “*not being motivated*” data were excluded. The total number of data epochs used in this study is 1873 epochs from the “*being motivated*” case that resulted in 1429 remembered (RR) and 444 forgot (RF) cases.

### 2.2. Cognitive Motivation Effect Identification

**Figure 1:**
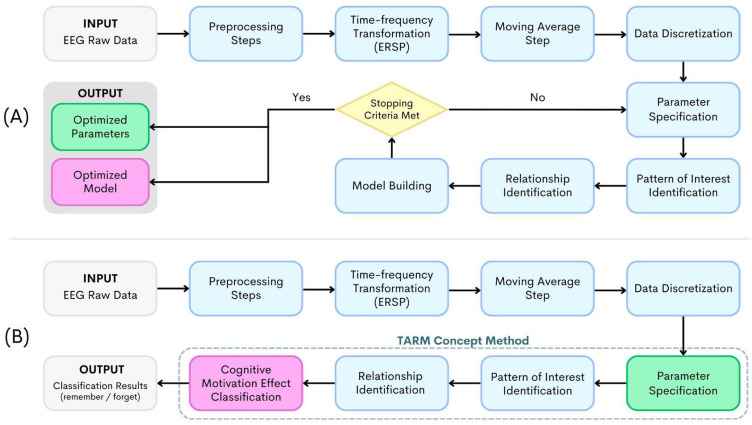
The processes of the cognitive motivation effect identification method proposed in this study: (**A**) the model construction part and (**B**) the identification part.

To identify the effect of motivation on brain activities related to cognitive performance, we designed the TARM-based method for EEG. Figure 1 shows the overview of the method’s process. The processes are divided into two parts: the model construction part, shown in Figure 1A, and the identification part, shown in Figure 1B. For the model construction part, the EEG data are preprocessed to remove physiological noise, power line noise, and eye-blinking artifacts. Only the epochs without saccade characteristics are used for analysis. Because alpha desynchronization is known from the previous study [16] to involve cognitive motivation, the preprocessed EEG epochs are transformed into ERSP data to prepare for determining desynchronization trends. The ERSP data are moving averaged to smoothen the spectral perturbation and to reveal the trend. Then, the trend data are represented by discretized sequences. The details of the preprocessing steps are described in Section 2.2.1. The discretized sequences are then analyzed to determine the temporal relationship between two signals; the details are explained in Section 2.2.2. The TARM concept is applied in this method to find the temporal relationship patterns. The temporal relationship patterns are used to build classification models for classifying the cognitive performance (whether the scene is remembered or forgotten); the details are given in Section 2.2.3. However, EEG signals are highly complex; the method requires some parameters along the processing pipeline to be optimized to obtain an effective classification model for identification. Section 2.2.4 describes the process for parameter and model optimization. The output parameter set and classification model are then used in the identification part. Figure 1B illustrates the procedure for the cognitive motivation effect identification part.

#### 2.2.1. Preprocessing Steps

In this study, the EEG signals were analyzed by MATLAB R2014b (MathWorks, Natick, MA, USA) with the open-source toolbox EEGLAB v13.4.4b [24]. MATLAB is a commercial software that allows complex matrix manipulation and computation, which is suitable for EEG signal processing. EEGLAB, which is the open-source toolbox working on the MATLAB environment, provides comprehensive tools from visualization, processing, and analysis to in-depth self-coding for specific studies. The preprocessing steps start with mapping the EEG signals to their corresponding electrode locations on the head model. Then, the average referencing was conducted by subtracting the average potential of all electrodes from each electrode at each time point. After that, both 0.5–50 Hz bandpass and 60 Hz notch filters were applied to remove physiological and power line noise, respectively. The signals were then marked into epochs and labeled by the participants’ motivation choices and recognition results regarding the scene stimuli. An epoch that has a signal voltage higher than 500 microvolts (μV) or lower than −500 microvolts was considered an abnormal value epoch and, thus, excluded from this study. The components related to eye-blinking artifacts were analyzed using the Independent Component Analysis (ICA) method and then selected and removed manually using the GUI tool of the EEGLAB toolbox. After that, the epoch with the saccade characteristic was manually selected and discarded. The epoch without response in the recognition test was also excluded. Finally, there are 1094 RR and 332 RF case epochs in this study.

All remaining epochs were transformed into the time-frequency domain using the Event-Related Spectral Perturbation (ERSP) method [25]. The average ERSP data across the alpha band (8–12Hz) were used in this study. Note that the preprocessing steps up to this point are the same as in our previous study [16]. Previously, we found that at the FCz and P3 electrodes the continuous alpha desynchronization patterns of the RR cases are significantly longer than those of the RF cases. Hence, the processed ERSP data from the FCz and P3 electrodes were used as representatives for attention and memorization areas, respectively, in this study.

**Figure 2:**
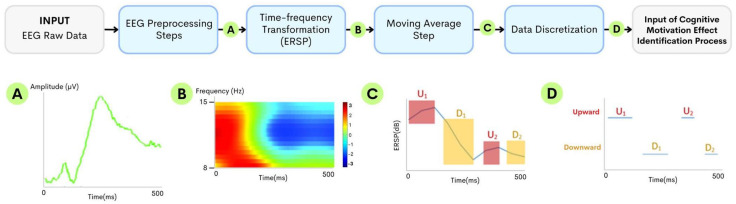
[**Top**] The preprocessing steps from EEG to discretized sequences with [**Bottom**] the examples of data output from each step: (A) EEG segment, (B) ERSP, (C) smoothened alpha band (8−12 Hz) ERSP, and (D) discretized trend sequences.

To reduce the complexity of the preprocessed ERSP data, the Simple Moving Average (SMA) method was applied. The trends were revealed. Nevertheless, a suitable SMA parameter should be selected. This is one of the parameters to be optimized later. The data were then discretized into sequences representing downward and upward trends. The downward trend indicates the continuous desynchronization period, while the upward trend relates to the synchronization period of the ERSP data. Each trend was represented by the starting and ending time points. The data sequence is considered to have a downward trend when all time points have continuously lower values than the preceding points. Similarly, the data sequence with continuously higher values than the preceding points is considered an upward trend. In this study, the downward trends of the FCz and P3 electrodes were analyzed as the potential attention and memorization sequences of interest. The processes of preprocessing EEG data into discretized sequences for the cognitive motivation effect identification method are shown in Figure 2. The bottom part of Figure 2 illustrates the examples of data output from each of the processes.

#### 2.2.2. Relationship Identification

The alpha desynchronization is known to relate to the attention state [26,27]. Based on the findings in our previous study [16], the continuous alpha desynchronization patterns of the RR cases are significantly longer than those of the RF cases. In this study, we hypothesized that motivation could lead the participant to continue paying attention to the stimulus, which, in turn, resulted in stimulus memorization. It is anticipated that, for the remembered cases, the attention downward sequences should occur before the memorization downward sequences. According to Allen’s 13 temporal logics [28], there are 3 possible temporal relationships between attention and memorization sequences based on this hypothesis: *before*, *contain*, and *overlap*. We explored the potential of these 3 temporal relationships for the association rules between brain areas.

**Figure 3:**
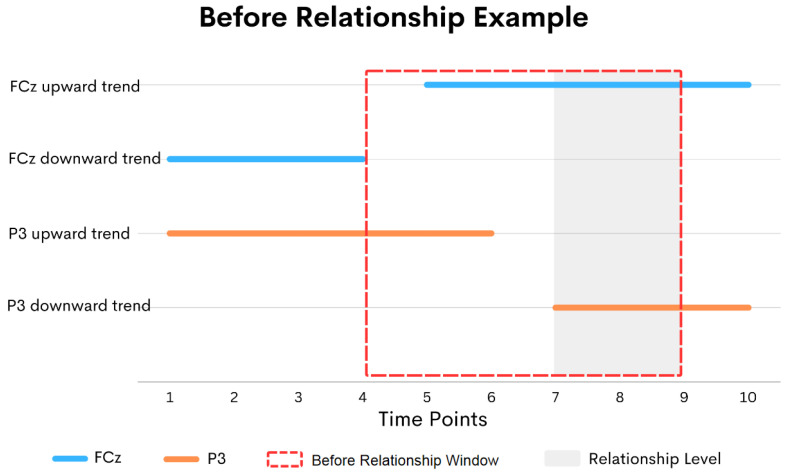
An example of before relationships.

For each relationship, we measured the relationship level, which indicates the likelihood that the relationship is related to the motivation that leads to the stimulus memorization. For the before relationship, the first (attention) sequence must start and end before the second (memorization) sequence starts. The longer the interval between the two sequences, the less likelihood that the second sequence is the result of the first sequence. We set a threshold window indicating that the two sequences are related. If the interval between the two sequences is within the threshold window, the two sequences are considered related. Hence, its relationship level is the ending time of the first sequence plus the length of the threshold window and then minus the starting time of the second sequence. An example of the before relationship in our study between the attention sequence (FCz downward trend) and the memorization sequence (P3 downward trend) can be presented in Figure 3. It should be noted that the suitable threshold window length is unknown; it is another parameter to be optimized later.

**Figure 4:**
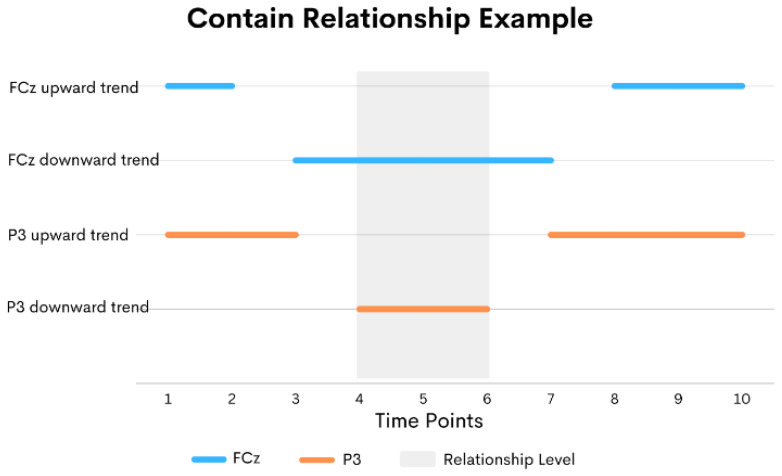
An example of contain relationships.

**Figure 5:**
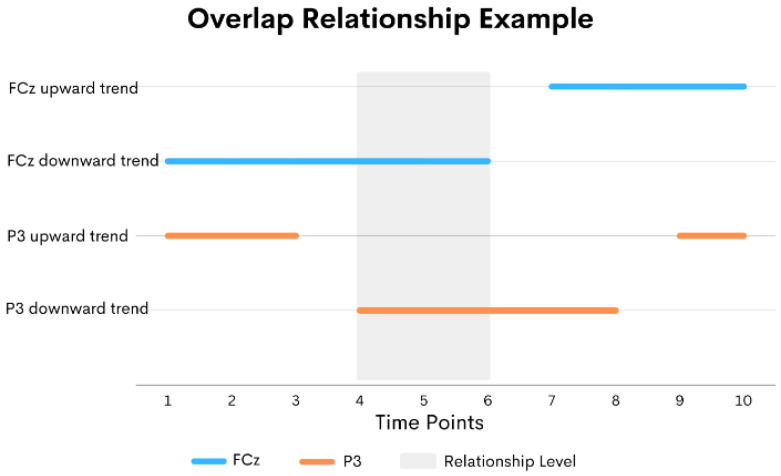
An example of overlap relationships.

For the contain relationship, the first sequence starts before the second sequence but ends later than the ending of the second sequence. The relationship level of the contain relationship is the length of the second sequence. Figure 4 shows an example of a contain relationship. Lastly, in the overlap relationship, the first sequence must also start before the second sequence. The first sequence ends after the second sequence starts but before the second sequence ends. The relationship level of this overlap relationship is the ending time of the first sequence minus the starting time of the second sequence. An example of the overlap relationship is shown in Figure 5.

The discretized sequences of epoch data from the FCz and P3 electrodes are determined for these temporal relationships. An epoch can comprise multiple temporal relationships. Therefore, the influence of each of these relationships must be considered together. In this study, the method based on the concept of TARM was used to analyze the cognitive motivation effect through these relationships.

#### 2.2.3. The Application of Temporal Association Rule Mining Concept

Temporal Association Rule Mining (TARM) [19] is used to identify the pattern of relationship among items that occurred within the data while considering the temporal constraint. Several parameters are required to consider the confidence of the identified rule. The support threshold is used to consider whether the item has occurred frequently enough to be considered an item of interest. The temporal support threshold is used to consider whether the relationship between two items occurred for sufficiently long enough to be considered as having a relationship of interest. Lastly, the confidence of the relationship is indicated by the confidence value.

In this study, we applied the TARM concept to identify the temporal relationships between attention and memorization as the effect of motivation resulting in stimulus memorization. The attention downward sequences and memorization downward sequences are the items to which we direct interest. The alpha desynchronization trend should continue for a sufficient duration to be considered a pattern. The support threshold is the minimum length of the downward sequence that is considered a pattern; the sequence shorter than the support threshold is discarded. To our knowledge, the suitable sequence length is unknown. Because the suitable sequence length for attention could differ from that for memorization, we used 2 support thresholds to determine the FCz and P3 downward sequences separately; they are called the attention and memorization pattern length thresholds, respectively.

The temporal support threshold is the sufficient period of time that the attention downward sequences relating to memorization downward sequences are considered to have a relationship; a higher relationship level than the temporal support threshold is counted as a relationship. There are 3 temporal support thresholds, one for each of the temporal relationships described in Section 2.2.2. They are called the before-relationship length threshold, contain-relationship length threshold, and overlap-relationship length threshold.

**Figure 6:**
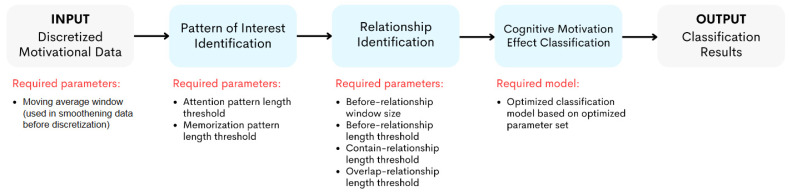
The processes to identify the cognitive motivation effect. The parameters and model required to be optimized for each step were listed under each corresponding red label.

Based on the TARM concept, this method has 5 threshold values to be designed. When including the moving average parameter and the before-relationship window size, mentioned in Section 2.2.1 and Section 2.2.1, there are 7 parameters to design in order to identify the association relationships between attention and memorization. Table 1 includes all 7 method parameters and their descriptions. Figure 6 shows the processes at which the required parameters of this method are located.

The method filters the discretized downward sequences from the FCz and P3 electrodes to remove the sequences shorter than the attention and memorization pattern length thresholds. The FCz downward sequence that is longer than the attention pattern length threshold is considered an attention sequence. Likewise, the P3 downward sequence longer than the memorization pattern length threshold is considered the memorization sequence. Then, the temporal relationships of all attention and memorization sequences are analyzed. The relationship with a lower relationship level than the corresponding threshold is discarded. Finally, the remaining relationships are considered temporal relationships between attention and memorization resulting from being motivated.

Due to the complexity of the brain, it is anticipated that the combination of temporal relationships will be involved in cognitive motivation. We introduced the use of a classification model built from the “*being motivated*” epochs to evaluate how well the combination of temporal relationships could accurately identify whether the motivation could lead to stimulus memorization. We used 6 features as input for the classification model, including the occurrence number and relationship level of before relationships, the occurrence number and relationship level of contain relationships, and the occurrence number and relationship level of overlap relationships. The relationship occurrence numbers are related to how often that attention resulting in memorization occurred while being motivated. The relationship level, as described in Section 2.2.2, indicates the likelihood that the relationship is related to the motivation that leads to the stimulus memorization.

The classification model construction can be chosen from a variety of methods. In this study, we demonstrated our method with Support Vector Machine (SVM) as an example; the “templateSVM” MATLAB function with auto kernel scale Radial Basis Function (RBF) kernel was used. The output classification model from the method can be used to predict whether the EEG input data acquired while being motivated can lead to stimulus memorization afterward.

#### 2.2.4. Method Parameter and Model Optimization

The 7 method parameters could affect the accuracy of the identification method. To find the suitable values of these parameters and the acceptable classification model, the Artificial Bee Colony (ABC) algorithm was used. The ABC algorithm is a population-based metaheuristic optimization algorithm introduced by Karaboga [22]. The method is known for its simplicity and flexibility in implementation and combination with other algorithms. With these advantages, ABC was used as an example of a metaheuristic algorithm for the model construction part of our method. The ABC method was inspired by the intelligent foraging behavior of honeybees. The employed bees search for the positions of food sources while remembering the position for future foraging. Information on the food source is shared with the onlooker bees waiting at the hive. The onlooker bee selects its target according to the quality of the food source and searches for food sources in the target direction. When the employed bees and onlooker bees cannot find a better food source, they abandon the food source and become a scout exploring in a random direction for a new food source. In the ABC algorithm, onlooker and employed bees perform the search in the specific search space (exploitation), while scouts perform the wide exploration.

**Figure 7:**
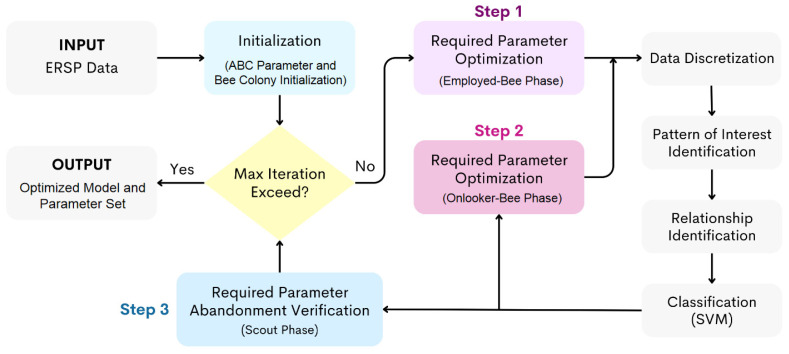
The detailed optimization part of the cognitive motivation effect identification method implemented with the ABC algorithm.

Figure 7 illustrates the processes to optimize the parameter set and obtain the optimized classification model; the processes in Figure 7 are related to Figure 1A but show more details of how ABC was incorporated. The search space for the bees is 7 dimensions for 7 parameters. The employed bee positions are randomly initialized. Then, the method proceeds to perform a set of ABC iterations and terminates when the number of iterations exceeds the specified maximum iteration. For each iteration, the employed-bee, onlooker-bee, and scout-bee phases were performed to find a good parameter set. The TARM-based processes used the best parameter set to identify temporal relationships and build an SVM classification model. The accuracy of the classification model was used as the food source quality.

Anuar et al.’s study [29] performed the ABC colony size tests ranging from 4 to 200 and suggested that the colony size should be at least 24. This study used a colony size of 28, which is 4 times the parameter numbers. The 28 bees comprised 14 employed and 14 onlooker bees. The maximum iteration number was set to 200. The range of each parameter was set based on the knowledge of our previous study [16] as 0 to 9 for the moving average window; 30 to 100 ms for attention and memorization pattern thresholds; 150 to 200 ms for before window size; and 30 to 100 ms for before-, contain-, and overlap-relationship length thresholds.

In the employed-bee phase, each employed bee performs a search near its employed food source. In the onlooker-bee phase, each onlooker bee selects an employed-bee food source position (parameter set) as the reference position to explore nearby areas. The selection is based on the probability function weighed on the quality (classification accuracy) of each food source [22]. A better-quality food source probably attracts more bees to exploit it. The employed and onlooker bees search for a nearby food source with the same calculation as mentioned in [22] but with different ranges. This study used 1/5 and 1/3 of the nearest neighboring food sources as the search range for employed and onlooker bees, respectively. Cosine similarity was used to identify these nearest neighboring food sources. If the quality of the new food source is better than the previous position, the bee position is updated. After both the employed-bee and onlooker-bee phases are finished, the scout phase is started. In the scout phase, the number of times each food source has been visited was counted. If any food source is visited exceeding a specified threshold, the employed bee will abandon its position and become a scout, randomly selecting a new position.

By applying the ABC algorithm, the optimized parameter set required for the identification method was obtained. However, there could be a bias problem resulting from the difference in the data of each stimulus category during the classification model-building process. In this study, the numbers of epochs in two motivated cases (RR and RF) are different. This situation is especially common when the experimental categories are based on the participants’ preferences, which can be varied and cannot be controlled. Using the unequal numbers of data from each category for training and testing of the SVM method could lead to obtaining a biased classification model. To avoid this problem, an equal number of data from each cognitive motivation case were randomly selected as the training and testing set. A total of 200 training data were used in the model-building process: a hundred from the RR epochs and another hundred from the RF epochs. Another 50 random epochs were selected from the leftover data, 25 from each category, to be used as the test set.

Because there is no known knowledge about the temporal relationship between attention and memorization, the objective value that determines the quality of an ABC food source is the classification accuracy of the model from the test set. Nevertheless, the different sampled training and test sets can result in different classification accuracy and food-source quality. In turn, the resulting parameter set can be different from one repetition to another, or the same parameter set can give different classification accuracy from one sample set to another. This problem could lead to an inaccurate selection of the optimized parameter set.

To ensure that the optimized parameter set obtained from the method can be used effectively in real applications in the future, we applied the Central Limit Theorem (CLT) [23] to mitigate the problem of variant outputs due to sampling variation. The problem resulted from the different sample sets when the sampling process was used in the optimization search process. With CLT, the method can identify the mean of population accuracy as the representative. CLT is a probability theory that states that “as the sampling number increases, the mean sampling distribution will increasingly become closer to a Gaussian distribution”. These Gaussian or normal distribution data are concentrated around the mean. In other words, with sufficiently large unbiased sample numbers from the whole population (with a finite level of variance), the mean of all samples from the same population will be (approximately) equal to the mean of the whole population. In this situation, the mean or median is suitable to be used as the representative of the overall sampling data and can also be used in exchange. In CLT, it is not feasible to state the exact sample size sufficient for a general approximation. However, at least 30 sample sizes are often suggested to produce an approximately normal sampling distribution from a non-normal parent distribution [30]. Additionally, in case the population is normally distributed, it is believed that if sampling at least 10 times, the sampling mean is able to assume a normal distribution [31,32]. In this study, the normal distribution cannot be assumed due to the possibility of varying relationship numbers and their temporal periods in each epoch. For our data, the number of relationships and their temporal period of a relationship could be affected by the other relationships within a limited attention period of 3 s. The longer a relationship, the lesser the temporal period available for the others. From the results in [30] and the suggestion in [31,32], the sample sizes of 10 and 50 are used as the comparison cases.

In this study, we applied the concept of CLT to identify the population median of the classification accuracies as the representative of the food source in the ABC algorithm. The CLT concept was applied through the implementation of the repeated sampling training and test set within the model-building process. Each sampling has its own model and accuracy. The median from all the repeated resulting accuracies was used as the representative quality of the food source in the ABC search process. Any food source with a Median Absolute Deviation (MAD) higher than 0.03 is regarded as an unstable food source and excluded from the quality update of the search process.

## 3. Results

This section presents the performance of the TARM-based method to identify the cognitive effect of motivation with only two electrodes, FCz and P3. The results of ABC with SVM, ABC with 10 times repeated sampling SVM (ABC-10RSVM), and ABC with 50 times repeated sampling SVM (ABC-50RSVM) were compared to find the best parameter set and cognitive motivation identification model. These parameters can be used as guidelines to configure parameters in the preprocessing steps, preparing the data before the model classification process. Then, the temporal relationship features were analyzed. Finally, the generalization performance of the best model was demonstrated using the data of individual participants. The accuracy of our result model is validated to be 74.5% on average with individual tests.

### 3.1. Cognitive Motivation Effect Classification Model Results and Parameter Set Suggestion

A set of seven parameters is required to be optimized. These parameters could affect the performance of the motivation effect identification model. These parameters include the moving average (MA) window, attention, and memorization pattern thresholds, the before-relationship window threshold, the before-relationship length threshold, the contain-relationship length threshold, and the overlap-relationship length threshold. We tested each of the three ABC-applied methods for 10 rounds to identify their average classification accuracies. These average classification accuracies will then be used as their performance representative. For the representative classification accuracy of the parameter set (from each round of the optimization), the median accuracy from 1000 random train and test sets was used. Since a large number of random samples (1000) were tested for each parameter set, the distribution of the results for each parameter set can be assumed to be a normal distribution according to CLT. The median accuracy from the accuracy results of all sampling is then suitable to be used as the representative accuracy for each of these parameter sets. These median accuracies for 10 times ABC optimization are presented in Table 2. Additionally, detailed information on the best model and parameter set of each method is presented in Table 3.

With the results in Table 2, the results of the ANOVA test, comparing the 10-round median accuracies from 16-participant sampling data of ABC-SVM, ABC-10RSVM, and ABC-50RSVM methods, suggested that the classification accuracy of the ABC-SVM method is significantly different from both ABC-10RSVM and ABC-50RSVM, with a P-value lower than 0.01. Additionally, the results between ABC-10RSM and ABC-50RSVM are not significantly different (*p*-value = 0.7771). The standard deviation results are 5.38%, 5.30%, and 5.18% for ABC-SVM, ABC-10RSVM, and ABC-50RSVM, respectively. The ABC-50RSVM method has the lowest variance among tested methods. All of the best model results for each method have an accuracy variance lower than 5%, as presented in Table 3. Additionally, the ANOVA test of the SD variance comparison among the methods resulted in no significant difference. The suggested parameters from ABC-10RSVM and ABC-50RSVM are almost the same except for the contain-relationship threshold parameter, as presented in Table 3.

Our results suggest that applying CLT to the ABC-SVM method can help us mitigate the problem of variant outputs due to sampling variation. This improvement can leverage the accuracy of the motivation effect identification model from our ABC-SVM with the TARM-based method. Additionally, with the lowest variance and highest classification accuracy, we suggested ABC-50RSVM for the cognitive motivation effect identification method. The best model has a confidence of 80% classification accuracy. Please also be aware that the 50 sampling number is not the magic number, and the number could vary depending on the data distribution. However, even with the lower mean classification accuracy (75.5% vs. 75.8%) and higher variance (5.30% vs. 5.18%) of the ABC-10RSVM to the ABC-50RSVM method, the difference in both cases is not statistically significant. With these results, it can be suggested that ABC-10RSVM can be used in the case of limited time constraints in which lowering model-building execution time is required and a slight variance classification accuracy is acceptable. Lastly, our parameter set suggestion is based on the results from 10 rounds of ABC for each method for the scenic stimulus, which may not represent the general use in real-world applications. With this problem, we suggested applying the ABC-RSVM process to build the model and identify the suitable parameter set before the application is used for the first time. A case of validation for the general use of the best model will also be presented later in this study.

From Table 3, the suggested parameters from ABC-50RSVM are a moving average window equal to 4, 50 ms for attention pattern length threshold (FCz downward), 30 ms for memorization pattern length threshold (P3 downward), 190 ms for before window size threshold, 50 ms for before-relationship length threshold, 60 ms for contain-relationship length threshold, and 30 ms for overlap-relationship length threshold. From this parameter set, we attempted to identify the potential relationship feature that is highly distinguished by the effect of motivation. These relationships could lead to the improvement of preprocessing steps and model building in the later study.

The results of our study also give evidence that motivation influences the activities within the brain differently in “being-motivated-and-remembered” and “being-motivated-but-forgot” cases. This influence is affecting the brain activities related to FCz and P3 electrodes, which are related to attention and memorization areas within the brain. From the knowledge of previous studies [13,14,15,16], we assumed the relationships from the attention sequence occurred before the memorization sequence. With this assumption, the results in this study give evidence that the brain activities in the attention area lead to the activities in the memorization area by a top-down process while being influenced by the effect of cognitive motivation. Unfortunately, the specific difference between brain activities of RR and RF cases cannot be known from the results of our method. The problem is due to our classification model using a combination of features from the attention and memorization sequences to identify the effect of motivation. Unlike linear SVM, which can summarize data with a set of parameters with fixed size (the weight coefficient), the transformation of SVM with RBF kernels is based on the pairwise distances between the training points resulting in the number of parameters growing with the size of the support vector, which makes it a non-parametric method. Therefore, the importance of each relationship characteristic (i.e., the weight coefficient in SVM) cannot be directly identified. However, the study tries to explore how motivation affects brain activities differently by identifying the importance of a specific feature in differentiating RR and RF cases in the next section.

### 3.2. Potential Feature Suggestion

To identify the difference between brain activities of RR and RF cases, we performed a statistical test comparing RR and RF cases of each suggested classification model input feature from the previous section. Because the temporal period of one relationship could be affected by the temporal period of other relationships within a limited 3 s temporal attention period, for example, the longer period of an overlap relationship leads to the shorter remaining temporal period available for other relationships. Therefore, the relationship features were not in the normal distribution. The Wilcoxon signed-rank test was used. The results of this test are presented in Table 4. Note that the average relationship-level feature of each relationship is not the input feature of our classification model. The average relationship levels represent the mean relationship level of the relationship for each epoch. They were only used to statistically analyze the difference between RR and RF in this section.

From the observation of our SVM input data, the before relationship is the pattern that is always found in motivated cases; from 200 epochs of each case, all 200 RR epochs and 198 RF epochs have a before relationship. There are 143 RR epochs and 135 RF epochs with the contain relationship. Lastly, there are 187 RR epochs and 174 RF epochs with an overlap relationship. This study hypothesized that the effect of motivation is affected by the combination of multiple relationships in each epoch. However, the fact that before-relationship patterns are found in most epochs could suggest that the occurrence of the before relationship could be highly influenced by the state of being more motivated than other relationships. In concordance with this observation result, the results of Table 4 also suggest that only the “average before-relationship level” is significantly different between RR and RF cases; the average before-relationship levels of RR cases are higher than those of RF cases. These results suggest that the brain activities of the RR cases had a memorization pattern that occurred following the ending of the attention pattern sooner than in the RF cases. However, the difference is at a 0.1 significant level, with a p-value equal to 0.07, so we suggested exploring the conditions that could affect this occurrence in more detail before using this knowledge in future studies.

**Figure 8:**
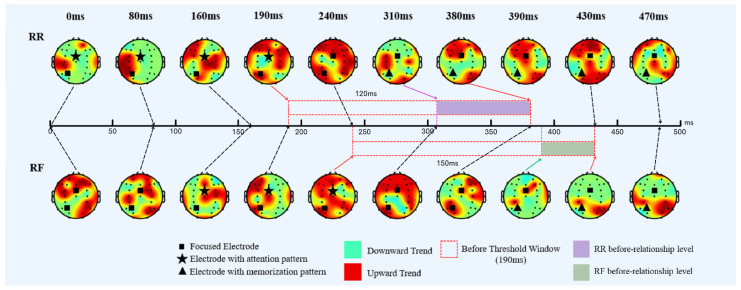
An example of temporal ERSP trend data of an RR epoch with a before relationship. The arrow marked the occurrence time of the corresponding head model.

As additional information, this study presents examples of before relationships in RR and RF cases in Figure 8. The examples are in the form of alpha frequency trend data (upward and downward) mapped onto all electrodes of the sequential temporal period head models. In Figure 8, the square symbol (■) represents the focused electrodes used in this study, which are FCz and P3. The pentagram symbol (★) represents the FCz electrode while having an attention pattern of interest, which is an attention pattern that leads to memorization. The triangle symbol (▲) represents the P3 electrode while having a memorization pattern of interest, which is the memorization pattern resulting from being motivated and having attention. The dotted red box during 190–310 ms for RR or 240–380 ms for RF represents the period between the ending of the attention pattern and the start of the memorization pattern mentioned in the previous paragraph. Since the before-threshold window has a fixed constant value, the distance between these two patterns is the counterpart of the before-relationship level. The higher before-relationship level is equal to the lower distance between the patterns. Figure 8 also shows that the RR case has a longer alpha desynchronization pattern than the RF case, as mentioned in the results of our previous work [16].

### 3.3. Model Validation

Lastly, we verified the generalization of the suggested model with the individual participant data. Since the RF case usually has a low number of epochs, only participants with RF epochs higher than 25 were tested. The model was tested with data from six participants, each with 1000 test sets. A test set comprised 20 randomly selected epochs from the RR and RF cases each. The results of this test are summarized and presented in Table 5.

The results suggest that our model can use only FCz and P3 electrodes to identify the effect of motivation with 70.65% classification accuracy on average. The model can classify the effect of being motivated acceptably for most participants, except for participant number 6, whose classification accuracy result is lower than the others. This may be due to age; brain alpha frequency becomes higher with age [33]. Participant number 6 is 33 years old, while the others are in their 20s. We tested the model with the data of another participant who is 37 years old but who has a number of RF epochs lower than 25; this participant was excluded from the validation participants. Because the number of RF epochs for this participant is very low, we used five RR epochs and five RF epochs for the test set. A classification accuracy of 53.11 percent was returned. The results indicate that the identification model can be used for users in their 20s; separate models should be constructed for different age ranges. Considering only participants in the 20s age range, the model resulting from the proposed method can be used to identify the effect of motivation on the individual participants with 74.5% mean classification accuracy.

## 4. Discussion and Conclusions

With the random scene selection in the experiment, the motivation leading to memorization could be the result of various motives. Some scenes may have objects that interest the participant as the motive [1]. Some participants may have a motive to demonstrate their competence to gain a higher score than others [2,3,4]. Other motives could be that the scenes have a unique artistic value that piques the curiosity of the participant or looks complex and makes the participant feel challenged to remember them [5]. Even for the same individual, the motive could be different given the change in time and situations. These differences in motives can affect the various brain areas [6,7,8,9,10,11,12], which, in turn, results in high numbers of EEG electrodes being required to identify the effect of motivation. Table 6 concludes the motives related to cognitive motivation and the affected electrodes resulting from existing EEG studies in the literature. In total, 28 electrodes would be required to measure all listed motives.

Instead of focusing on the motives, we proposed to shift the focus to the effect of motivation; the idea could allow us to use a smaller number of electrodes to measure in real education applications. The low number of electrodes can help lessen the burden on users and improve flexibility when used in a real-world environment. We previously found that motivation can affect both attention and memorization [13,16] and that attention leads to memorization [14]. In this study, we investigated the effect of motivation through the temporal relationship between the associated brain areas with two EEG electrodes representing attention and memorization.

Using the proposed method, the cognitive motivation identification model can identify the effect of motivation related to the cognitive performance of participants with 74.5% accuracy when validated with individual participant test sets. The model can be used for users in the 20s age range. Due to the complexity of human brains, the sampling process for data can lead to the problem of variant output values. This study used CLT to address this problem. The CLT-applied ABC-RSVM model from this method returned the acceptable parameter set in Table 3 for analyzing the temporal relationship patterns among the associated brain areas. Based on the acceptable parameter set, the results indicate that when the participant is motivated, attention precedes memorization, with a higher average before-relationship level in the RR cases than in the RF cases.

The findings of this study can be applied to help complement studies on the characteristics of visual stimuli that lead to motivation [34,35,36,37,38,39] for later use in educational applications. In educational applications, teaching materials are usually intended not only to motivate the student but also to make sure that their key points can later be remembered. By applying our model for the stimulus that was found to motivate the participants, the predicted results of whether the stimulus will likely be remembered afterward can be used to evaluate and help improve the teaching material in the future. Additionally, the conventional cognitive tests (e.g., Newcastle Spatial Memory Test [13]) used in education studies are usually performed without brain activity measurement. There could be various effects that influence the cognitive results of the participant (e.g., information missing due to the passage of time). However, using our method to identify the cognitive results based on brain signals at the learning event can help in evaluating the stimulus and teaching materials more efficiently by reducing the other influences between the event and the testing time. By applying the knowledge from this study, the learning application can adjust the stimulus (e.g., teaching materials) at the time of learning to be more suitable for personalized learning services.

There are some limitations to this study. The participants are sampled from Asian populations, which may not represent the general populations of other races. The age of the users could also influence the performance of the application due to the difference in alpha frequency with age [33]. The effect of age range and population race (e.g., Asian, African, and European) are also topics of interest for future in-depth studies to address this limitation. When more data are collected to cover different races and age ranges, we suggest applying our method to identify the suitable parameter set and to construct the identification model. Note that as the method in this study intended to identify the effect of motivation with reduced EEG electrode requirements, the method cannot identify the motive of their motivation during their learning process. The method requires data from motivated participants as inputs to identify their cognitive performance. Therefore, the motivation of the user has to be identified before the process of this study. The method is suggested to be used with the stimulus that is known to motivate the participant.

## Figures and Tables

**Table 1:** The method parameters of cognitive motivation effect identification based on the TARM concept.

Method Parameter Name	Related Process	Description
Moving average window	Moving average process	To smoothen the spectral perturbation and reveal the potential hidden trend
Attention pattern length thresholds	Pattern of interest identification	To identify the attention sequence that has a sufficient alpha desynchronization trend
Memorization pattern length thresholds	Pattern of interest identification	To identify the memorization sequence that has a sufficient alpha desynchronization trend
Before-relationship window size	Relationship identification	To identify the before relationship and calculate the before-relationship level
Before-relationship length thresholds	Relationship identification	To identify the before relationship resulting from the effect of motivation and leading to stimulus memorization
Contain-relationship length thresholds	Relationship identification	To identify the contain relationship resulting from the effect of motivation and leading to stimulus memorization
Overlap-relationship length thresholds	Relationship identification	To identify the overlap relationship resulting from the effect of motivation and leading to stimulus memorization

**Table 2:** The median accuracy results of 1000 sampling test sets for each parameter set for motivation effect identification method.

Methods	Classification Accuracy										
	1st	2nd	3rd	4th	5th	6th	7th	8th	9th	10th	Average
**ABC-SVM**	**73%**	71%	66%	72%	70%	72%	71%	72%	72%	72%	70.8%
**ABC-10RSVM**	74%	76%	**80%**	78%	71%	75%	75%	73%	75%	78%	75.5%
**ABC-50RSVM**	74%	73%	76%	**80%**	75%	76%	74%	77%	77%	76%	75.8%

The bold number is the highest accuracy of the method.

**Table 3:** The detailed information on the best results from each parameter and optimization model.

Methods	Window		Threshold (ms)					Accuracy (%)					
	MA (pts)	Before (ms)	Pattern A	Pattern B	Relationship			Min	Max	SD	Mean	Med	[Q1, Q3]
					Before	Contain	Overlap						
**ABC-SVM** **(Round 1)**	2	200	30	90	50	100	40	58	86	4.35	72.93	73	[70, 76]
**ABC-10RSVM** **(Round 3)**	4	190	50	30	50	30	30	66	91	3.99	79.8	80	[77, 82.5]
**ABC-50RSVM** **(Round 4)**	4	190	50	30	50	60	30	69	91	3.96	80.39	80	[78, 83]

**Table 4:** Wilcoxon signed-rank test result between RR and RF cases of each feature.

Features	Z	Asymp. Sig. (2-Tailed)
**Before-relationship occurrence number**	−0.906 ^p^	0.365
**Before-relationship level**	−0.219 ^p^	0.827
**Average before-relationship level**	−1.809 ^n^	0.070
**Contain-relationship occurrence number**	−0.668 ^n^	0.504
**Contain-relationship level**	−1.71 ^p^	0.864
**Average contain-relationship level**	−1.206 ^p^	0.228
**Overlap relationship occurrence number**	−0.845 ^n^	0.398
**Overlap-relationship level**	−1.103 ^n^	0.270
**Average overlap-relationship level**	−0.311 ^p^	0.756

^p^: Based on positive ranks, ^n^: based on negative ranks.

**Table 5:** The results of individual test sets with the best model from the ABC-50RSVM method.

Participant	Accuracy (%)		
	Median	Mean	SD
**1**	85	85.44	5.51
**2**	80	81.35	6.11
**3**	70	70.78	7.52
**4**	70	70.60	6.96
**5**	65	64.27	7.32
**6**	50	51.47	7.79

**Table 6:** The affected EEG electrodes of motivation effect resulting from motives.

Motives	Author/Year	Affected Electrodes	Affected Electrode Number
Reward	Ven et al., 2016 [9]	C3, Cz, C4, CP1, CP2, P3, Pz, and P4	8
Challenge	Ma et al., 2017 [10]	F4, F6, F8, FC4, FC6, and FT8	6
Interest	Jin et al., 2015 [11]	F1, Fz, F2, FC1, FCz, FC2, C1, Cz, C2, CP1, CPz, CP2, P1, Pz, and P2	15
Curiosity	Brydevall et al., 2018 [12]	Fpz, AFz, Fz, FCz, and Cz	5
All listed motives	-	F1, F2, F4, F6, F8, FC4, FC6, FT8, Fz, FC1, FCz, FC2, Fpz, AFz, C1, C2, C3, C4, Cz, CP1, CP2, CPz, Cz, P1, P2, P3, P4, and Pz	28

## Data Availability

The data used in this study are the same as our previous study [16]; the dataset is described and available in our data article [40]. The code of our methods can be found at https://drive.google.com/drive/folders/1S-_L8-zReu_AIUE6a_YoRHyQh_f67Y3Y (accessed on 5 April 2024).

## Abbreviations

The following abbreviations are used in this study:

ABC	artificial bee colony
ABC-SVM	ABC with SVM
ABC-10RSVM	ABC with 10 times repeated sampling SVM
ABC-50RSVM	ABC with 50 times repeated sampling SVM
CLT	central limit theorem
ERSP	event-related spectral perturbation
ICA	independent component analysis
MA	moving average
MAD	median absolute deviation
RBF	radial basis function
RF	being motivated but forgot
RR	being motivated and remembered
SVM	support vector machine
TARM	temporal association rule mining
